# Hepatoprotective Effect of Carob Pulp Flour (*Ceratonia siliqua* L.) Extract Obtained by Optimized Microwave-Assisted Extraction

**DOI:** 10.3390/pharmaceutics14030657

**Published:** 2022-03-17

**Authors:** Nikola Martić, Jana Zahorec, Nebojša Stilinović, Bojana Andrejić-Višnjić, Branimir Pavlić, Nebojša Kladar, Dragana Šoronja-Simović, Zita Šereš, Miodrag Vujčić, Olga Horvat, Aleksandar Rašković

**Affiliations:** 1 Department of Pharmacology, Toxicology, and Clinical Pharmacology, Faculty of Medicine, University of Novi Sad, 21000 Novi Sad, Serbia; nikola.martic@mf.uns.ac.rs (N.M.); nebojsa.stilinovic@mf.uns.ac.rs (N.S.); miodrag.vujcic@axonsm.com (M.V.); olga.horvat@mf.uns.ac.rs (O.H.); aleksandar.raskovic@mf.uns.ac.rs (A.R.); 2 Faculty of Technology, University of Novi Sad, 21000 Novi Sad, Serbia; bpavlic@uns.ac.rs (B.P.); dragana@tf.uns.ac.rs (D.Š.-S.); zitas@tf.uns.ac.rs (Z.Š.); 3 Department of Histology and Embryology, Faculty of Medicine, University of Novi Sad, 21000 Novi Sad, Serbia; bojana.andrejic-visnjic@mf.uns.ac.rs; 4 Department of Pharmacy, Faculty of Medicine, University of Novi Sad, 21000 Novi Sad, Serbia; nebojsa.kladar@mf.uns.ac.rs; 5 Axon Site Management, Veselina Masleše 32, 21000 Novi Sad, Serbia

**Keywords:** carob pulp flour, microwave-assisted extraction, antioxidant activity, response surface methodology, paracetamol-induced hepatotoxicity, CYP2E1

## Abstract

To examine antioxidant capacity and the hepatoprotective effect of carob pulp flour, microwave-assisted extraction was performed. The influence of ethanol concentration (0–40% *w*/*w*), extraction time (5–25 min) and irradiation power (400–800 W) on DPPH, FRAP and ABTS antioxidant activity of carob pulp flour extract was evaluated. The strongest influence was that of the ethanol concentration, followed by extraction time. Optimal process parameters for maximizing total antioxidant activity were determined, using response surface methodology: ethanol concentration 40%, time 25 min and power 800 W. Carob extract obtained at optimal conditions (CE) was analyzed in vivo using a paracetamol-induced hepatotoxicity model in mice. Treatment with CE attenuated the parameters of liver injury, especially aspartate and alanine aminotransferase activity, and prevented paracetamol-induced increase in malondialdehyde levels. Pretreatment with CE reversed the activities of superoxide dismutase, catalase, glutathione peroxidase and glutathione S-transferase enzymes after the high dose of paracetamol in the liver. Hepatotoxicity induced using a toxic dose of paracetamol was also seen through histopathological alterations, which were significantly reduced in the groups treated with CE prior to paracetamol. Still, the number of Kupffer cells and macrophages did not differ among groups. Finally, pretreatment of mice with CE and paracetamol significantly decreased the expression of cytochrome P450 2E1 (CYP2E1) in hepatocytes.

## 1. Introduction

Cultivation of the carob tree (*Ceratonia siliqua* L.) is mostly correlated to the Mediterranean region but the production of carob is widely spread throughout the world [1]. Industrial processing of carob fruit is focused primarily on the production of locust bean gum from endosperm of carob seeds, which is used as a thickener and stabilizer in the food industry, and is used in other industries such as cosmetics, pharmaceuticals and textiles [2,3]. After the separation of seeds from carob fruit there remains a significant amount of carob pulp, which is most often used as animal feed [4]. However, in recent years, carob pulp has been the focus of many studies related to the use of carob in human nutrition [5,6]. Furthermore, carob pulp has been recognized as a valuable source of polyphenols.

Polyphenolic compounds have been considered as an important class of bioactive compounds known for their antioxidant properties and beneficial effects on chronic diseases and ageing [7]. Therefore, a lot of research has been performed in order to determine and characterize the content of polyphenols in carob pulp flour [8,9,10] and their health impact [11]. Gallic acid was found to be the dominant compound in carob flour extracts, followed by (–)-epigallocatechin, (–)-epigallocatechin gallate and (–)-epicatechin gallate [12]. However, the concentration of total polyphenols in carob flour strongly depends on genetics, the environment and the extraction method [13]. Therefore, it is essential to adjust the extraction technique and to optimize its conditions in order to maximize the yield of target compounds.

The isolation of antioxidants from natural sources is commonly achieved using solid–liquid extractions, which require a long time and have limitations regarding mass transfer. Therefore, novel extraction techniques such as microwave-assisted extraction (MAE) have been introduced in order to overcome the mentioned disadvantages [14] and to provide better efficiency for the recovery of bioactive compounds from various sources. The main factors influencing MAE are temperature, extraction time, irradiation power, matrix properties and solvent (type, concentration and polarity) [15]. Therefore, these variables, as well as properties of target compounds, should be taken into account for the optimization of MAE process.

Optimization of extraction processes is often performed using a one-factor-at-a-time approach, which implies that the influence of independent variables on chosen responses is investigated one by one. This can be time-consuming and expensive, but also ineffective, since this approach does not provide any insight about the effects that can be a result of interactions between variables. For this reason, as an adequate tool for the optimization of processes in which a dependent variable is influenced by several input factors, response surface methodology (RSM) can be successfully applied [16]. Previously, RSM has been used for the optimization of the extraction of polyphenols from different plant materials, while antioxidant activity of different extracts obtained by MAE was also recently studied [17,18,19,20]. Furthermore, the full utilization of the extraction conditions optimization could be found in in vivo animal models. Namely, the carob pulp extracts are already tested for antioxidant properties in various animal models. Still, the extracts administered in those experiments were made without any optimization procedures employed [21,22].

Therefore, the aim of the present work was to investigate the effects of MAE conditions (extraction time, ethanol concentration and microwave irradiation power) on the antioxidant properties of carob pulp flour extracts and to apply RSM in order to optimize these conditions for obtaining the highest antioxidant activity of obtained liquid extracts determined by DPPH, FRAP and ABTS assays. In order to bring the obtained extract into effective action another goal of this study was to examine its protective properties in paracetamol-induced hepatotoxicity in mice.

## 2. Materials and Methods

### 2.1. Plant Material

Carob (*Ceratonia siliqua* L., the family of *Leguminosae* (*Fabaceae*)) pulp flour (CF) used for extraction was a commercial product cultivated in Croatia and processed (deseeded, ground, and roasted), packed and sold by Aroma začini, D.O.O. (commercially available in Serbia).

### 2.2. Chemicals

Folin–Ciocalteu reagent, (±)-catechin, 1,1-diphenyl-2-picryl-hydrazyl-hydrate (DPPH•), 2,2′-azino-bis (3-ethylbenzothiazoline-6-sulphonic acid) diammonium salt (ABTS), Trolox (6-hydroxy-2,5,7,8-tetramethylchroman-2-carboxylic acid) and 2-p-iodophenyl-3-p-nitrophenyl-5-phenyl tetrazolium chloride (INT) were purchased from Sigma (Sigma-Aldrich GmbH, Steinheim, Germany). Gallic acid and paracetamol were purchased from Sigma-Aldrich (St. Louis, MO, USA). Potassium persulfate (99% pure) was obtained from Acros Organics (Geel, Belgium). All other chemicals used were of analytical reagent grade.

### 2.3. Microwave-Assisted Extraction

Microwave-assisted extraction (MAE) was performed in a remodeled commercial microwave oven (NN-E201W, Panasonic, Osaka, Japan) into which a reflux condenser balloon was placed. For each experimental run, 10.0 g of plant material was mixed with 100 mL of corresponding solvent of water and ethanol. The parameters used as independent variables for the optimization of MAE process were: ethanol concentration (0, 20 and 40% *w*/*w*), extraction time (5, 15 and 25 min) and irradiation power (400, 600 and 800 W). After cooling obtained extracts were centrifuged and filtered. Extracts were collected into glass flasks and stored at 4 °C until further analyses.

### 2.4. Antioxidant Activity

#### 2.4.1. DPPH Assay

Free radical scavenging activity of carob pulp extracts towards 2,2-diphenyl-1-picrylhydrazyl free radicals (DPPH•) was assessed following the procedure of Espín et al. [23]. A solution of DPPH reagent in methanol (65 µM) was freshly prepared and adjusted with methanol to achieve absorbance of 0.70 (±0.02). Then, 2.9 mL of DPPH reagent and 0.1 mL of properly diluted extracts were mixed in the 10 mL cuvettes and incubated for 60 min at ambient temperature. Absorbance was measured in three replicates at 517 nm (6300 Spectrophotometer, Jenway, Stone, UK). The calibration curve was obtained by measuring free radical scavenging of freshly prepared Trolox aqueous solutions (0–0.8 mM, *R*^2^ = 0.999). The obtained results were reported as µM of Trolox equivalents per g dry weight (DW).

#### 2.4.2. FRAP Assay

Reducing the power of extracts towards Fe^3+^ was determined according to assay previously described by Oyaizu [24]. The FRAP reagent was freshly prepared from 300 mM acetate buffer (pH = 3.6), 10 mM 2,4,6-tris (2-pyridil)-s triazine (TPZT), 40 mM HCl solution and 20 mM FeCl_3_ aqueous solution, which were mixed in 10:1:1 (*v*/*v*/*v*) ratio. 1.9 mL of FRAP reagent and 0.1 mL of properly diluted extracts were mixed and incubated at 37 °C in the dark for 10 min. Absorbance was measured in three replicates at 593 nm (6300 Spectrophotometer (Jenway, Stone, UK)). Calibration was performed using freshly prepared Fe^2+^ (Fe_2_SO_4_) aqueous solutions (0–0.23 mM, *R*^2^ = 0.999). The obtained results were reported as µM of Fe^2+^ equivalents per g DW.

#### 2.4.3. ABTS Assay

The scavenging capacity of extracts toward ABTS free radicals was measured using a method previously reported by Re et al. [25], with some modifications. ABTS stock solution was freshly prepared from mixture of 1:1 (*v*/*v*) aqueous solutions of 2.45 mM K_2_S_2_O_8_ and 7 mM ABTS (2,2′-azino-bis- (-3-ethylbenzothiazoline-6-sulfonic acid) diammonium salt) and left for 16 h in the dark. Prepared ABTS stock solution was diluted with 300 mM acetate buffer (pH = 3.6) until an absorbance of 0.70 (±0.02) was achieved. ABTS reagent (2.9 mL) and properly diluted extracts (0.1 mL) were mixed and incubated for 5 h in the dark at room temperature. Absorbance was then measured in three replicates at 734 nm (6300 Spectrophotometer, Jenway, Stone, UK). To obtain the calibration curve freshly prepared Trolox aqueous solutions (0–0.8 mM, *R*^2^ = 0.989) were used. Results were expressed as µM of Trolox equivalents per g DW.

### 2.5. Experimental Design and Statistical Analysis

The response surface methodology (RSM) was applied in order to investigate the impact of MAE parameters on investigated responses and was used for the MAE process optimization. The experiments were conducted according to the Box–Behnken experimental design where three variables were investigated on three levels: ethanol concentration (0–40% *w*/*w*), extraction time (5–25 min) and irradiation power (400–800 W). The experiment consisted of seventeen randomized runs with five replicates at the central point. In order to avoid the relevance of the units of process variables, the values were coded in range from −1 to 1 (Table 1).

The chosen responses were fitted to the second-order polynomial model:

(1) $$Y=\beta_{0}+\overset{3}{\underset{i=1}{\sum }}\beta_{i}X_{i}+\overset{3}{\underset{i=1}{\sum }}\beta_{\mathrm{ii}}X_{i}^{2}+\sum^{\;}\overset{3}{\underset{i<j=1}{\sum }}\beta_{\mathrm{ij}}X_{i}X_{j}$$

where Y represents the response variable, X_i_ and X_j_ are the independent variables affecting the response, and β_0_, β_i_, β_ii_ and β_ij_ are the regression coefficients for intercept, linear, quadratic and interaction terms, respectively.

Optimal extraction conditions were determined considering antioxidant activity parameters as responses. Treatment of multiple responses was based on the desirability function (D), which was also used for the selection of optimal MAE conditions [26]. Statistical testing was performed by analysis of variance (ANOVA) with two levels of significance: 0.01 (highly significant) and 0.05 (significant). The adequacy of the chosen model was assessed according to the coefficient of multiple determination (*R*^2^), coefficient of variance (CV) and *p*-values for the model and lack of fit testing. The experimental design and multiple linear regression analysis were performed using Design-Expert v.11 Trial (Stat-Ease, Minneapolis, MN, USA).

### 2.6. Chemical Characterization—Phenolic Profile

Carob pulp extract obtained at optimal conditions (CE) was analyzed using chemical characterization and quantification of the selected compounds. The compounds of interest were separated on Nucleosil C18 column (250 mm, i.d. 4.6 mm, 5 μm particle size; Macherey Nagel) and analyzed by HPLC-DAD Agilent Technologies 1100 liquid chromatographer (Agilent Technologies, Santa Clara, CA, USA). Gradient elution was applied according to the following program: 0 min–10% B; 10 min–25% B; 20 min–45% B; 35 min–70% B; 40 min–100% B; 46 min–10% B, where solvents A and B were methanol and 1% (*v/v*) aqueous solution of formic acid, respectively. The mobile phase variable flow rate according to the following program was applied: 0–10 min, 1 mL/min; 10–20 min, 0.8 mL/min; 20–30 min, 0.7 mL/min; 30–46 min, 1 mL/min, while the volume of sample injection was 10 µL. For the purpose of phenolic compounds’ quantification in the evaluated extracts, chemical standard substances of caffeic acid (CA); gallic acid (GA); *p*-coumaric acid (pQA); *trans*-cinnamic acid (CNA); rosmarinic acid (RA); chlorogenic acid (CHA); ferulic acid (FA); quercetin (Qe); rutin (R) and quercitrin (Qt) were analyzed under the same experimental conditions. The chromatograms were monitored at 280 nm (GA, CA and CNA), 330 nm (pQA, CHA, RA, FA and Qe) and 350 nm (R and Qt), while the quantity of phenolic compounds was expressed as mg per g of dry extract [27].

### 2.7. Experimental Animals and Ethical Statement

Healthy, adult, male Swiss-Webster mice, 8 weeks old with average body weight of 32 ± 3 g were used in this study. The animals were brought from the Military Medical Academy (Belgrade, Serbia) and kept in Ehret Uni-Protect cabinets with a High Efficiency Particulate Air (HEPA) filter system (EHRET Labor- und Pharmatechnik GmbH & Co. KG, Emmendingen, Germany). The animals were kept in polycarbonate transparent cages with a standard circadian cycle (12 h day–12 h night), controlled temperature (22–25 °C), relative humidity (55 ± 1.5%) and free access to pellet food and water (the food was removed from the animals only 12 h before and 6 h after the paracetamol was administered). All experimental procedures and animal care have been carried out in accordance with the ethical principles outlined by the EU Directive 2010/63/EU on animal welfare and under the Law of Animal Welfare of the Republic of Serbia (OG RS 41/09). The animal study was approved by the Ethics Committee for the protection of the welfare of laboratory animals by the University of Novi Sad (Novi Sad, Serbia; No. 04-81/2-1) and the approval of the Ministry of Agriculture, Forestry and Water Management of Republic of Serbia was obtained (Belgrade, Serbia; No. 323-07-00884/2020-05).

### 2.8. In Vivo Experimental Design

The experiment was performed on a total of 42 mice that were randomly divided into 6 groups of 7 animals. All mice received treatments through oral gavage in accordance with the following protocol:

- ConS—saline 1 mL/kg, seven days p.o.;
- ConP—saline 1 mL/kg seven days p.o. +1 single dose of paracetamol 110 mg/kg p.o.;
- CE100—carob extract 100 mg/kg for seven days p.o.;
- CE100 + P—carob extract 100 mg/kg for seven days p.o. +1 single dose of paracetamol 110 mg/kg p.o.;
- CE200—carob extract 200 mg/kg for seven days p.o.;
- CE200 + P—carob extract 200 mg/kg for seven days p.o. +1 single dose of paracetamol 110 mg/kg p.o.

In order to induce hepatotoxicity animals were treated with paracetamol. Before use paracetamol was dissolved in saline with a temperature of 60 °C under constant stirring, and before administration chilled to 37 °C. After 24 h of paracetamol administration the animals were sacrificed in order to gather blood samples of a sufficient quantity for biochemical tests as well as liver tissue samples for examining oxidative status parameters. In addition, a histological assessment was performed.

### 2.9. Liver Function Tests

The serum samples were used to measure the enzymatic activity of aspartate (AST, EC 2.6.1.1) and alanine (ALT, EC 2.6.1.2) aminotransferase, direct bilirubin and the concentrations of urea, creatinine and uric acid. Spectrophotometric methods were employed for biochemical analyses, using the automatic analyzer AU480 (Beckman Coulter Inc., Indianapolis, IN, USA). All samples were analyzed by well-established spectrophotometric methods using commercially available kits and in accordance with the supplied instruction manuals [28].

### 2.10. Determination of In Vivo Antioxidant Activity

The oxidative status in the liver was determined by measurement of lipid peroxidation (LP) level and activities of oxidative stress enzymes, including total superoxide dismutase (T-SOD, EC 1.15.1.1); catalase (CAT, 1.11.1.6); glutathione peroxidase (GPx, 1.11.1.9); glutathione reductase (GR, 1.6.4.2) and glutathione S-transferase (GST, 2.5.1.18) in liver homogenates. All measurements were completed by spectrophotometric methods. Liver tissues were homogenized at 4 °C in a ratio 1:3 (*w/v*) with TRIS–HCl buffered solution. Oxidative stress parameters and lipid peroxidation were analyzed from these liver homogenates. Measurements were performed in duplicate for each sample. LP intensity was measured indirectly by the quantity of malondialdehyde (MDA), a final product of lipid breakdown caused by peroxidation damage [29]. The determination of specific activity of T-SOD is based on the reaction of xanthine with xanthine oxidase, whereby a superoxide anion radical is formed, which reduces the oxidized cytochrome c. The reduction rate is monitored spectrophotometrically at 550 nm [30]. The CAT activity was determined by decomposition rate of H_2_O_2_ at 240 nm [31]. The activities of GR and GPx were based on quantifying the decrease of absorbance caused by the oxidation of NADPH at 340 nm [32,33]. GST catalyzes the conjugation reaction of 1-chloro-2,4-dinitro-benzene (CDNB) with the thiol group of glutathione. The resulting CDNB-S-glutathione conjugate was measured at an absorption maximum of 340 nm [32].

### 2.11. Histopathology and Immunohistochemistry Assessment

The histological assessment was blind, performed by two researchers by light microscopy. The histological analysis was performed on a small piece of liver tissue sampled from each animal. Samples were fixed in Bouin’s solution for 24 h. After that, samples were dehydrated in a graded series of isopropyl alcohol and embedded in paraffin blocks. For each mouse, four consecutive 5 µm thick tissue sections were cut, using a rotation microtome (Sakura Finetek USA, Inc., Torrance, CA, USA). Two sections were stained with routine hematoxylin and eosin (H&E) and Periodic Acid Schiff (PAS) method. The two remaining sections underwent the immunohistochemical procedure of staining, where, in accordance with the manufacturer’s instructions, antibodies Iba-1 (1:8000, AB178847, Abcam, Cambridge, UK) and CYP2E1 (1:200, CSB-PA006425EA01H4, Flarebio, College Park, MD, USA) were applied. Histological analysis was performed under Olympus BX-43 light microscope (Olympus, Tokyo, Japan) with an attached Olympus DP 73 video camera (Olympus, Tokyo, Japan). The free image software Image J (version 1.51h, National Institute of Health, Bethesda, MD, USA) was used for further morphometric analyses of Iba-1 and CYP2E1 stained slides. Iba-1 staining marked the macrophages of the liver. Based on 5 high-power field (HPF, 40× magnification) photography, the average percentage of Iba-1 positive cells (Iba1+) was calculated. CYP2E1 antibody detects cells with cytochrome P450 2E1 isoenzyme activity. The fraction of liver tissue with CYP2E1 positivity was measured using Image J software on 5 HPF photographs of each tissue slide.

## 3. Results and Discussion

### 3.1. Model Adequacy

The Box–Behnken experimental design, developed for the optimization process considering antioxidant activity (DPPH, FRAB and ABTS) along with experimentally obtained values for each response under different MAE conditions, is presented in Table 1. Independent variables were chosen considering the results of previous research regarding the extraction of phenolic compounds from carob pulp [18,19] which are considered to be responsible for plant material antioxidant activity. The mentioned researchers suggested that using a lower L/S ratio, lower ethanol concentrations (up to 50%) and medium microwave irradiation power (400 to 800 W), carob extracts with higher yields of antioxidants are achieved.

Experimental results for individual responses were fitted to a second-order polynomial model (Equation (1)). In order to test the adequacy of obtained models the analysis of variance (ANOVA) was applied. Corresponding *p*-values of regression coefficients for each investigated response and descriptive statistics’ parameters are summarized in Table 2. As the first indicator of model fitness, the coefficient of determination (R^2^) was used. Particularly high R^2^ for DPPH, FRAP and ABTS (0.945, 0.848 and 0.922, respectively), suggested that all applied models were in accordance with obtained experimental results. Furthermore, the dispersion of the experimental data was evaluated by CV value. Since a small value of CV indicates low variation in the mean value [34], the relatively low CV (<12%) obtained for all responses suggested good reproducibility of the investigated systems. Moreover, the chosen model provided proper representation of experimental results, which was confirmed by the statistically significant *p*-values (<0.05) in model testing for all responses. With the exception of ABTS, lack-of-fit testing was insignificant (*p* > 0.05), therefore, the assumption of constant variance was satisfied.

**Figure 1:**
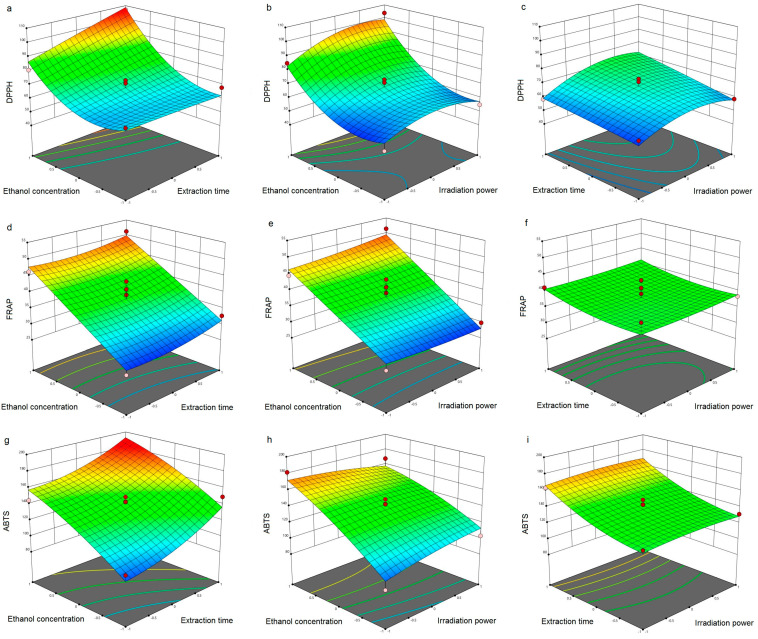
Response surface plots showing combined effect of investigated independent variables on: (**a**–**c**) DPPH; (**d**–**f**) FRAP; and (**g**–**i**) ABTS.

Since the adequacy of the applied models was confirmed, obtained experimental data were further analyzed using RSM in order to develop individual regression equations which would demonstrate the empirical relationship between the response values and applied extraction conditions. Multiple regression coefficients in Equation (1) were generated using the method of least squares (MLS). These calculations enabled the assembling of the predictive model equations (Equations (2)–(4)) for DPPH, FRAP and ABTS, respectively. Since the coefficients of variables with insignificant influence of corresponding responses could be neglected, following equations are presented as a reduced form of Equation (1):

(2) $$\mathrm{DPPH}=67.95+17.26X_{1}+5.14X_{2}+12.58X_{1}^{2}-7.15X_{3}^{2}$$

(3) $$\mathrm{FRAP}=37.95+9.60X_{1}$$

(4) $$\mathrm{ABTS}=142.66+30.18X_{1}+19.59X_{2}$$

where X_1_, X_2_ and X_3_ are ethanol concentration, extraction time and irradiation power, respectively. Furthermore, as a result of obtained regression equations, the three-dimensional surface plots for the investigated responses were created (Figure 1).

### 3.2. Antioxidant Activity of Carob Flour Extracts

The influence of MAE operational parameters on the antioxidant activity of CF extracts was evaluated using three in vitro model systems and the results are presented in Figure 1. Since plant polyphenols free the radicals by electron or hydrogen transfer reactions, DPPH and ABTS assays were used to measure the CF radical scavenging capacity. Polyphenols could also prevent oxidation by reduction of pro-oxidant metals and inhibition of oxidizing enzymes [35], therefore FRAP assay was used to measure the reducing power of obtained CF extracts.

The radical scavenging capacity of extracts obtained by MAE towards DPPH and ABTS radicals was from 49.87–106.85 µM TE/g DW and 85.73–185.64 µM TE/g DW, respectively (Table 1). The lowest values for the scavenging capacity of DPPH and ABTS radicals were observed when the same experimental conditions were applied: ethanol concentration 0% (water as solvent), extraction time 15 min and irradiation power 400 W (Table 1). On the other hand, extract obtained using 40% ethanol, 25 min and 600 W exhibited the highest scavenging capacity of both DPPH and ABTS radicals. In vitro antioxidant activity of carob pulp ethanol extracts obtained by MAE was already examined by DPPH [19], however the results were expressed as a percentage of DPPH inhibition, therefore it is not possible to compare them with our results. Roseiro et al. [36] compared the antioxidant capacity of carob pulp extracts obtained by conventional extraction techniques (shaking flasks and ultrasound extraction) and supercritical extraction. The results of their study showed lower antioxidant activity of the obtained carob extracts in comparison to our results, with the exception of supercritical extraction (0.130 mmol TEAC/g). On the other hand, the highest antioxidant activity (DPPH) of desugared carob pulp extracts, also obtained by supercritical extraction, did not exceed 550 µM TE/kg of carob pulp [37]. This could imply that the application MAE would significantly improve antioxidant capacity of CF, not only in comparison with conventional extraction but also with some modern extraction techniques. As far as authors are aware, the scavenging capacity of carob pulp flour extracts towards ABTS free radicals has not yet been investigated.

Reducing the power of CF extracts varied between 21.93 and 51.45 µM Fe^2+^/g DW (Table 1). The highest values for FRAP were observed under the following conditions: ethanol concentration 40%, extraction time 15 min and irradiation power 800 W. However, it should be emphasized that second highest value (less than 0.5% lower) for reducing power was observed in CF extract obtained under the same operational parameter that resulted in the highest DPPH and ABTS scavenging capacity (Run 11). No comparable data regarding the FRAP of carob pulp flour were found in the literature, however, the antioxidant activity of carob fruit extracts determined by FRAP were investigated by Goulas and Georgiou [38]. Their results indicated that the variety of carob fruit also has an influence on the antioxidant activity of carob fruit extracts obtained by ultrasound-assisted extraction, and may vary from 29.2 up to 339.7 mg FeSO_4_/100 g.

### 3.3. Optimization of MAE Process

One of the goals of this study was to optimize MAE parameters using RSM as the optimizing tool in order to enhance the antioxidant activity of carob flour extracts. Experimental results in the present study showed that ethanol concentration exhibited a highly significant (*p* < 0.01) positive single effect on all investigated responses (Figure 1), as well as significant (*p* < 0.05) influence on the quadratic term for DPPH (Table 2). Moreover, the abovementioned parameter was the only one of the investigated variables which influenced the FRAP antioxidant activity of CF extract (Figure 1). The presented results imply that a solvent with a higher concentration of ethanol is needed in order to maximize each individual response. Furthermore, extraction time had highly significant and a significant positive single effect on ABTS and DPPH, respectively, suggesting that it would be more desirable to use a longer extraction time. On the other hand, irradiation power had an insignificant single effect on antioxidant activity of obtained extracts (*p* > 0.05), however, in the case of DPPH significance of its quadratic term was observed (Table 2, Figure 1).

For optimization purposes, previously obtained second-order polynomial models were utilized for individual responses (DPPH, FRAP and ABTS). Optimization was carried out regarding total antioxidant activity as well and the corresponding results are presented in Table 3. In order to optimize all three responses at the same time, a desirability function of 0.900 was employed and optimized extraction conditions were estimated to be: ethanol concentration 40%, extraction time 25 min and irradiation power 800 W. Determined optimal conditions for MAE of carob flour confirmed the previously discussed results.

Furthermore, CE was used in the in vivo mice model to investigate its antioxidant and hepatoprotective potential.

### 3.4. Chemical Characterization—Determination of Phenolic Profile

**Figure 2:**
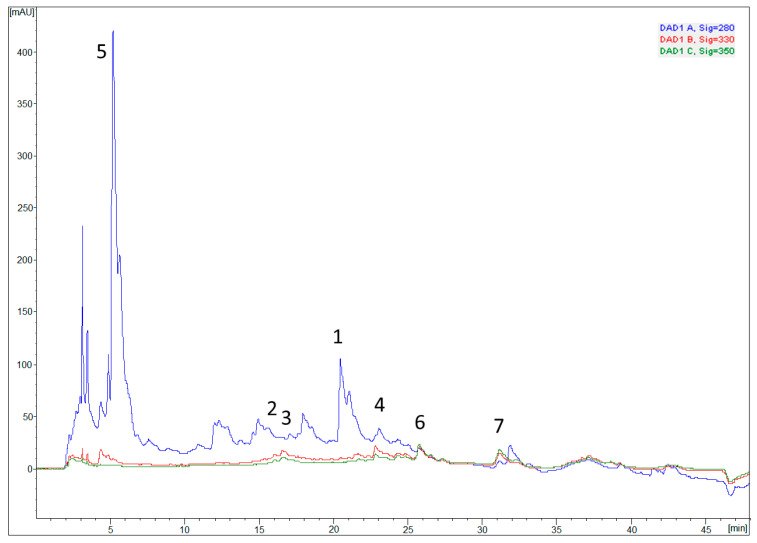
Chromatogram of optimized carob pulp extract with detection at 280, 330 and 350 nm. Identified compounds: 1—caffeic acid; 2—*p*-coumaric acid; 3—quercetin; 4—chlorogenic acid; 5—gallic acid; 6—rutin; 7—quercitrin.

Optimized carob flour extract (CE) was subjected to characterization of the phenolic profile (Figure 2), since they are considered to be responsible for antioxidant activity.

As shown in Table 4, 7 phenolic compounds were detected and quantified. It was determined that gallic acid was present in the highest share among the investigated phenolic compounds (0.46424 mg/g of dry extract), which is in accordance with literature data [19,38]. Other phenolic compounds identified in larger quantities were caffeic acid, quercitrin and p-coumaric acid in the descending order shown (Table 4).

The presented results can give insight into the phenolic profile of CE, as well as to determine the concentration of compounds, which surely contribute to the antioxidant activity of the extract itself, and that are assumed to be the carriers of pharmacodynamic effects.

### 3.5. Effects of Carob Flour Extract on Paracetamol-Induced Serum Biochemical Parameters

Carob extract hepatoprotective effects were examined by determining enzyme activities, and the markers of hepatocellular injury. It is already documented that high doses of paracetamol can cause a significant increase in ALT and AST levels [39]. The application of paracetamol toxic doses significantly increased the activity of ALT and AST in serum compared to the control (*p* < 0.05). In animals treated with carob flour extract in both doses (100 mg/kg and 200 mg/kg), the activities of transaminases were lower in comparison to the control. The pretreatment of animals with carob extract attenuates the parameters of paracetamol’s hepatotoxicity, especially for ALT and AST activity which were significantly lower in the groups CE100 + P and CE200 + P compared to animals treated with saline and paracetamol (*p* < 0.05). This was confirmed in the study performed on rats, with dextran as a hepatotoxic agent, where serum values of ALT and AST were statistically significantly lower in all groups treated with carob extract compared to the non-treated groups [22]. The intake of carob extract with paracetamol in both doses (100 mg/kg and 200 mg/kg) decreased the concentration of direct bilirubin compared to the control, although it was not significant (Table 5).

In addition, the nephroprotective properties of carob extract were also investigated. Urea concentrations showed no statistical significance between the groups. The intake of higher dose CE prior to paracetamol decreased the levels of creatinine compared to the ConP group. The intake of paracetamol with CE significantly decreased the concentration of uric acid in both groups (CE100 + P and CE200 + P) compared to animals of the ConP group (*p* < 0.05) (Table 5). These results suggest that CE has the potential to restore paracetamol-induced renal excretory dysfunction, which supports the findings of other, similar studies [40,41].

### 3.6. Effects of Carob Flour Extract on Paracetamol-Induced Oxidative Stress

**Figure 3:**
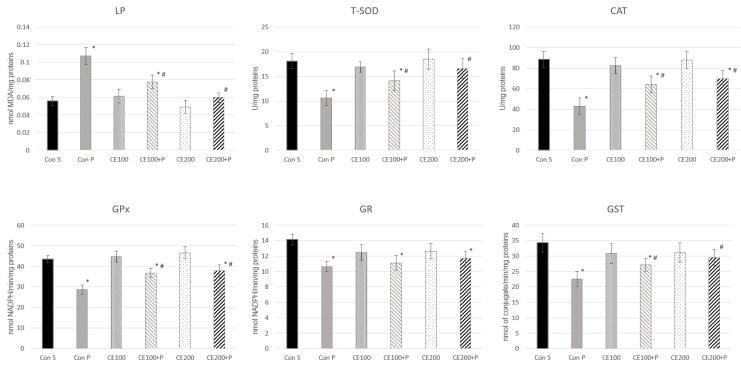
Effects of carob extract on paracetamol-induced oxidative stress (* *p* < 0.05 significantly different from ConS; # *p* < 0.05 significantly different from ConP).

To investigate the antioxidative potential of CE to prevent biochemical changes in the mice liver induced by toxic dose of paracetamol, LP was measured through the MDA level and activities of T-SOD, CAT, GPx, GR and GST as biomarkers of carob extract effects. It is well known that LP is known as one of the key mechanisms of cell injury caused by reactive oxygen species. MDA is one of the main secondary products of LP, used as a pointer of cell membrane injury [42]. Carob extract showed the ability to prevent increase in MDA levels induced by paracetamol, which suggests that CE can protect cellular integrity. This is supported by earlier studies that showed that the carob pods and leaf extract significantly decreases MDA levels and hepatic cells’ injury [43,44,45]. High doses of paracetamol increased the hepatic MDA level, when compared to the control, confirming the oxidative damage of the cell membrane (*p* < 0.05). Figure 3 demonstrated that in the group treated with CE and paracetamol, the MDA level was significantly decreased in both applied doses of CE. The activities of T-SOD, CAT, GPx, GR and GST enzymes were decreased in the liver homogenates of animals treated with paracetamol (ConP), compared to the control group with saline only (ConS). The treatment with CE in doses of 100 mg/kg and 200 mg/kg prior to paracetamol showed increased activity of T-SOD, CAT, GPx and GST enzymes compared to the ConP group, and these results are consistent with other studies, however, completed on different models [44,45]. The results of the present study showed that treatment with CE could regulate different parameters in liver homogenates, since it showed the capacity to reverse these parameters close to the values of the control group.

### 3.7. Histological, Immunohistochemical and Morphometric Analysis of Liver Tissue

As already mentioned, paracetamol, when used in toxic doses, can cause liver damage in experimental animals. Paracetamol in toxic doses causes severe centrilobular hepatic necrosis, which is proved in humans as well. The cause of hepatic histopathological alterations in paracetamol overdose are reactive metabolites, synthetised by liver cytochrome P450 2E1 (CYP2E1) and 3A4 (CYP3A4) enzymes. These toxic metabolites prevail over the detoxifying capacity of hepatocytes, which, through oxidative stress, eventually leads to hepatocyte damage.

**Figure 4:**
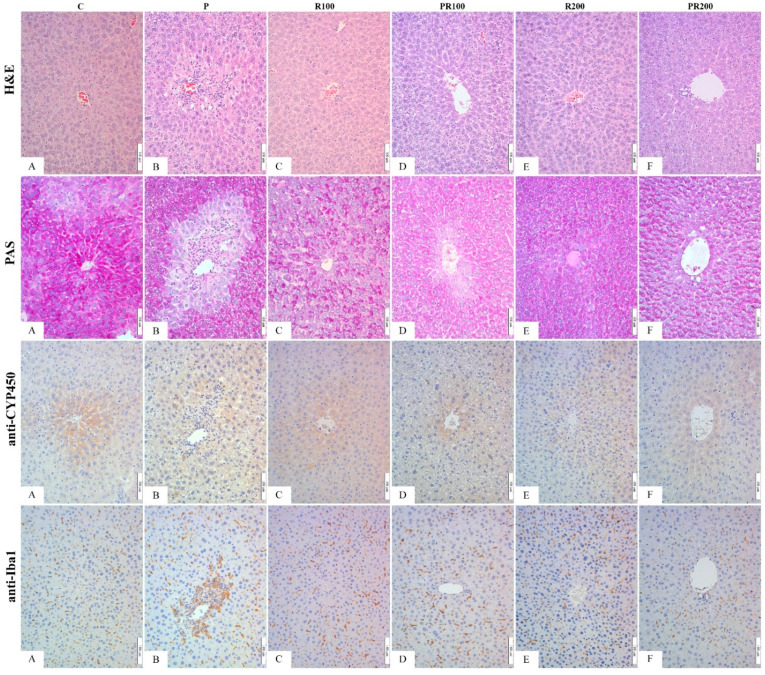
Histological and immunohistochemical analysis of liver tissue of healthy animals and animals with paracetamol-induced liver injury (40×, H&E—hematoxylin and eosin; PAS—Periodic acid-Schiff; anti-CYP450—monoclonal antibody, clone 2E1; anti-Iba1—monoclonal antibody). **Legend: H&E**—(**A**) normal liver histology of ConS group animals; (**B**) centrolobular necrosis and inflammatory infiltrate in ConP animals; (**C**) normal liver histology in CE100 animals; (**D**) moderate to mild parenchymatous degeneration in CE100+P animals; (**E**) unchanged liver histology in CE200 animals; (**F**) almost annulled paracetamol-induced changes (rare, solitary swollen hepatocytes) of CE200+P animals: **PAS**—(**A**) even distribution of glycogen in ConS group; (**B**) central depletion of glycogen in ConP group; (**C**) even, diffuse disptribution of glycogen of CE100 group; (**D**) mild centolobular depletion of glycogen in CE100+P group; (**E**) regular, diffuse glycogen distribution in CE200 group; (**F**) glycogen present in the level of the ConS group in CE200+P group. **anti-CYP450**—(**A**) smaller fraction of positive cells in cenrolobular areas of ConS group; (**B**) increased population of positive cells diffuselly trough lobule in ConP; (**C**) CE100 group has rare, centrolobular positive cells similar to ConS; (**D**) CE100+P showing centrilobular and periportal positive cells, but less than ConP; (**E**) rare, mildly positive, diffuse cells in CE200; (**F**) CE200+P showing rare centrilobular and periportal positive cells, less than ConP. **anti-Iba1**—(**A**) diffuse, solitary postive cells in ConS; (**B**) predominantly centrolobular positivity in ConP; (**C**) diffuse, solitary moderatelly postive cells in CE100; (**D**) diffuse, solitary strongly postive cells in CE100+P; (**E**) rare, diffuse, solitary mildly postive cells in CE200 (**F**) diffuse, solitary strongly postive cells in CE200+P.

Induction of oxidative stress is demonstrated through significantly increased LP, and change of antioxidant enzymes in the ConP group compared to the ConS [46]. In addition, histological examination revealed that the liver tissue of the ConS group had no evident changes in histological structure or cellular morphology. Hepatocytes are polygonal, with one (rarely two) nucleus, centrally placed in the cell. Hepatocytes form hepatic plates, radiating from the central vein to peripherally placed portal ares. Sinusoids running between the hepatic plates showed no signs of hyperemia or hemmorage. Inflammatory infiltrate was not present in lobules or portal areas (ConS/HE, Figure 4). PAS staining reveled adequate glycogen content in the hepatocytes of the control group (ConS/PAS, Figure 4). The tissue of ConP group animals, treated with toxic doses of paracetamol, showed a severe stage of parenchymatous degeneration, and damaged hepatocytes were present throughout most of liver lobule (ConP/HE, Figure 4). The cytoplasm of the hepatocytes was swollen and vacuolized, while the nuclei were smaller, irregularly shaped, and hyperchromatic. PAS staining revealed severely depleted glycogen in the hepatocytes, particularly of the central lobule area (ConP/PAS, Figure 4). Application of CE only, in both doses (100 mg/kg and 200 mg/kg), resulted in alleviation of the histological changes in liver histology, to the point that it was remarkably similar to the saline-treated animals of ConS group (CE100/HE, CE100/PAS, CE200/HE, CE200/PAS, Figure 4). Pretreatment with CE and toxic doses of paracetamol lead to improved liver histology compared to paracetamol-only treated animals. A dose of 100 mg of CE reduced the paracetamol-induced changes (moderate to mild parenchymatous degeneration and without or minimal glycogen depletion in hepatocytes) (CE100 + P/HE, CE100 + P/PAS, Figure 4). A dose of 200 mg/kg almost annulled the paracetamol-induced changes (rare, solitary perilobular hepatocytes were swollen, while glycogen was present in the level of the ConS group animals) (CE200 + P/HE, CE200 + P/PAS, Figure 4).

**Figure 5:**
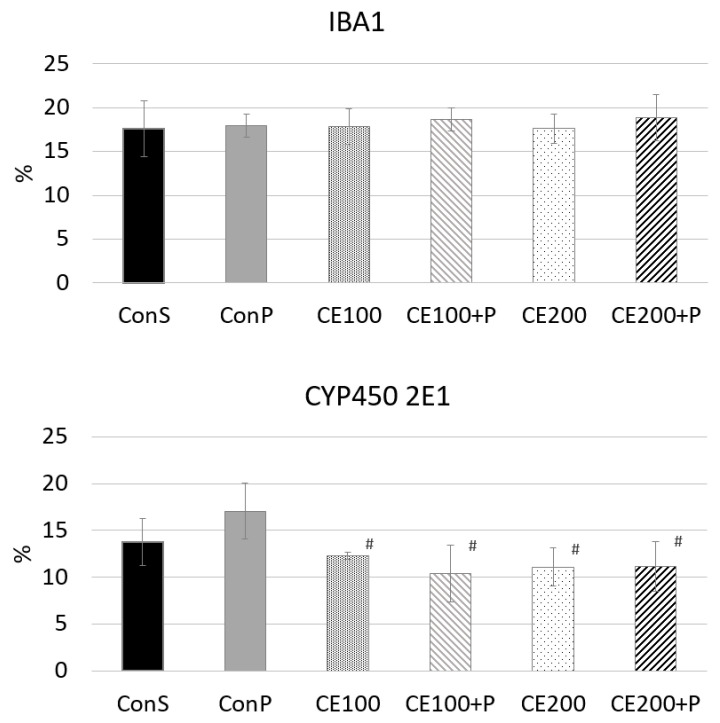
Morphometric analysis of liver tissue of healthy animals and animals with paracetamol-induced liver injury (# *p* < 0.05 significantly different from ConP).

Along with damaged hepatocytes, our results demonstrate a larger fraction of CYP2E1-positive hepatocytes in the ConP group compared to the ConS group, clearly due to the oxidative stress-related paracetamol effect, and increased production of toxic metabolites. This result is in accordance with other literature data. Many natural or pharmaceutical compounds were tested, in order to investigate its potential to prevent hepatotoxicity. Some of them reduced the induction of CYP2E1, the same as CE, which is interpreted as hepatoprotective [47]. In our study, the hepatoprotective effect of CE (in both doses), was demonstrated through reduction in the CYP2E1 induction in CE100 + P and CE200 + P groups (*p* < 0.05), as demonstrated in Figure 5. This is in line with a significant LP decrease in the CE100 + P and CE200 + P groups and is further confirmed through alleviation of histological changes.

While significant progress has been made in the understanding of intracellular signaling mechanisms of paracetamol hepatotoxicity, a considerable amount of debate still occurs in the literature over the role of sterile inflammation [48]. The most controversial question is whether the inflammatory response contributes to the injury or whether it is beneficial or even essential for survival, however that is beyond the scope of this research. Nevertheless, there has yet to be a definitive demonstration of a specific inflammatory cell that is directly responsible for cell death after paracetamol overdose. The most commonly associated cell type with cytotoxicity is the neutrophil, but both Kupffer cells and monocyte-derived macrophages have also been implicated in the paracetamol model [49]. The commonly measured markers of inflammation are increased serum levels of interleukins (IL-1ß and IL-18) and the presence of inflammatory cells. We investigated the presence of Kupffer cells and monocyte-derived macrophages as a percentage of Iba-1+ cells in the liver, and came to the conclusion that it does not differ among groups (Figure 5). It would be more precise to say that there are differences, but they are not statistically significant. The ConP group had slightly higher values compared to ConS, and it may point out the “direction” of changes in cellular composition of inflammatory infiltrate. Small variations in the presence of Iba1-positive cells may be seen among the CE100 and CE100 + P, and CE200 and CE200 + P, but once more, without statistically proven significance. It would be incorrect to claim that inflammation is not induced. There are two plausible explanations. The first of those is that macrophages and monocyte-derived cells are not the first line of defense after intoxication, which is also implied by other authors, who claim that pro-inflammatory mediators activate and recruit first the neutrophils and later monocytes [48]. In this study, animals were sacrificed 24 h after paracetamol application, so, indeed, this might not be enough time to induce and observe some statistically significant change in the presence of the Iba1-positive cells. The second explanation for an absence of Iba1 differences among groups may be explained based on the concept of M1/M2-macrophage polarization; M1-macrophages show high phagocytosis and cytotoxicity, whereas M2-macrophages may play a role in tissue repair and reparative fibrosis [50]. M1-macrophages are mostly activated in early phases of liver damage according to most studies [51,52]. M1 type of macrophages can be visualized by CD68. Regarding Iba1, some studies say that it marks M1, but others disagree [53]. It would be a more suitable marker of the M2 type, which is increased in later stages of tissue repair, and that could explain why we have not discovered any statistically significant differences 24 h after paracetamol application.

## 4. Conclusions

Based on the presented results of this study it could be concluded that RSM can be successfully used for the optimization of the extraction of carob flour antioxidants using microwave-assisted extraction, due to the satisfactory statistical parameters (*R*^2^ and CV) and ANOVA for the obtained models and lack of fit testing. Solvent (ethanol) concentration was designated as the most influential MAE parameter affecting the antioxidant activity of carob flour extracts, followed by the extraction time. Therefore, optimized conditions for obtaining CF extracts with maximized antioxidant activity by MAE were ethanol concentration 40%, extraction time 25 min and irradiation power 800 W. The pronounced antioxidant activity of the optimized extract was associated with its high content of gallic acid. Furthermore, the results of the in vivo part of the study indicate that carob flour extract inhibits an increase of the serum ALT and AST, as well as lipid peroxidation levels induced by paracetamol. Additionally, carob flour increases the enzyme antioxidant defense mechanisms present in the livers of mice. Finally, histological, immunohistochemical and morphometric analysis confirmed the ameliorative effect of carob flour extract in the present liver injury model. Overall, it could be concluded that carob flour as a raw material could be utilized for the extraction of valuable antioxidants and hepatoprotective agents.

## Figures and Tables

**Table 1:** Box–Behnken experimental design with natural and coded MAE conditions and experimentally obtained values of antioxidative activity (DPPH, FRAP and ABTS).

Run	Independent Variables			Investigated Responses		
	X_1_-Ethanol Concentration (%)	X_2_-Extraction Time (min)	X_3_-Irradiation Power (W)	DPPH (μM TE/g DW)	FRAP (μM Fe^2+^/g DW)	ABTS (μM TE/g DW)
1	40 (1)	15 (0)	400 (−1)	85.00	44.49	181.50
2	20 (0)	15 (0)	600 (0)	71.05	43.30	142.82
3	20 (0)	25 (1)	800 (1)	68.92	37.96	165.54
4	0 (−1)	5 (−1)	600 (0)	65.52	26.70	100.00
5	20 (0)	25 (1)	400 (−1)	58.37	41.04	163.39
6	40 (1)	15 (0)	800 (1)	103.79	51.45	167.69
7	40 (1)	5 (−1)	600 (0)	80.66	46.25	144.67
8	0 (−1)	25 (1)	600 (0)	67.98	32.65	149.12
9	20 (0)	5 (−1)	800 (1)	58.54	38.33	131.16
10	0 (−1)	15 (0)	800 (1)	54.89	29.81	103.23
11	40 (1)	25 (1)	600 (0)	106.85	51.22	185.64
12	20 (0)	5 (−1)	400 (−1)	56.25	41.07	131.16
13	20 (0)	15 (0)	600 (0)	65.09	40.94	142.79
14	20 (0)	15 (0)	600 (0)	64.58	35.17	135.77
15	20 (0)	15 (0)	600 (0)	65.77	31.19	143.02
16	20 (0)	15 (0)	600 (0)	73.26	39.14	148.90
17	0 (−1)	15 (0)	400 (−1)	49.87	27.44	85.73

**Table 2:** Analysis of variance (ANOVA) of the investigated responses.

Source	DPPH	FRAP	ABTS
	*p*-Value	*p*-Value	*p*-Value
Model	0.0013 *	0.0329 *	0.0040 *
X_1_-EtOH (%)	<0.0001 **	0.0005 **	0.0002 **
X_2_-Time (min)	0.0364 *	0.4286	0.0020 **
X_3_-Power (W)	0.0550	0.7869	0.8634
X_12_	0.0733	0.9149	0.7355
X_13_	0.2611	0.6194	0.2180
X_23_	0.4880	0.9706	0.9286
X_11_	0.0025 *	0.8676	0.3585
X_22_	0.9215	0.5723	0.2124
X_33_	0.0353 *	0.6381	0.6603
Lack of fit	0.1314	0.6249	0.0155 *
Mean value	70.38	38.71	142.48
Std. Dev.	5.64	4.43	11.57
CV	8.01	11.43	8.12
*R* ^2^	0.945	0.848	0.922

* significant (*p* < 0.05), ** highly significant (*p* < 0.01).

**Table 3:** Optimal MAE conditions for carob flour extraction.

Optimized Conditions	Ethanol Concentration (%)	Extraction Time (min)	Irradiation Power (W)
DPPH	40	22.4	400
FRAP	40	25.0	800
ABTS	40	23.7	600
Total antioxidant activity	40	25.0	800

**Table 4:** Chemical characterization of optimized carob pulp extract—phenolic profile.

Compound	Content (mg/g of Dry Extract) *
Caffeic acid	0.05353 ± 0.00268
*p*-coumaric acid	0.02386 ± 0.00239
Quercetin	0.00318 ± 0.00022
Trans-cinnamic acid	<LOD
Chlorogenic acid	0.01667 ± 0.00083
Rosmarinic acid	<LOD
Gallic acid	0.46424 ± 0.06964
Rutin	0.01883 ± 0.00151
Quercitrin	0.03967 ± 0.00198
Ferulic acid	<LOD

* The results were expressed as X ± SD, <LOD—not detected (LOD *trans*-cinnamic acid—0.00003 mg/g extract; LOD rosmarinic acid—0.00015 mg/g extract; LOD ferulic acid—0.00009 mg/g extract).

**Table 5:** Serum activity of alanine aminotransferase (ALT); aspartate aminotransferase (AST); direct bilirubin level; urea; creatinine; and uric acid (mean ± SD; n = 7) in animals treated with saline and carob extract.

	ConS	ConP	CE100	CE100 + P	CE200	CE200 + P
ALT (U/I)	68.00 ± 12.06	100.67 ± 8.39 ^a^	46.17 ± 12.98 ^b^	60.17 ± 18.02 ^b^	55.67 ± 10.35 ^b^	53.00 ± 7.90 ^b^
AST (U/I)	237.25 ± 72.06	348.00 ± 51.83 ^a^	187.80 ± 47.28 ^b^	204.67 ± 59.91 ^b^	193.33 ± 46.68 ^b^	190.83 ± 32.01 ^b^
Direct bilirubin	1.28 ± 0.31	1.45 ± 0.26	1.12 ± 0.08	1.17 ± 0.16	1.22 ± 0.10	1.14 ± 0.14
Urea (mmol/L)	7.12 ± 0.86	7.17 ± 1.94	7.18 ± 2.05	7.65 ± 1.22	7.28 ± 2.33	7.99 ± 0.42
Creatinine (μmol/L)	21.80 ± 2.28	24.00 ± 2.51	18.40 ± 3.65 ^b^	24.40 ± 2.61	19.33 ± 4.32	19.29 ± 2.43 ^b^
Uric acid (μmol/L)	181.00 ± 25.35	182.40 ± 27.40	174.00 ± 34.87	108.33 ± 24.81 ^a,b^	134.4 ± 30.21	63.57 ± 11.21 ^a,b^

^a^*p* < 0.05 significantly different from ConS group; ^b^ *p* < 0.05 significantly different from ConP group.

## Data Availability

The data presented in this study are contained within the article and Supplementary Material.

## Supplementary Materials

The following are available online at https://www.mdpi.com/article/10.3390/pharmaceutics14030657/s1, Table S1: Animal body mass (mean ± SD; n = 7) in animals treated with saline and carob extract.

## References

[1] Brand E. (1984). Carob. Nutr. Food Sci..

[2] Catarino F. (1993). The carob tree. An exemplary plant. Naturopa.

[3] Barak S., Mudgil D. (2014). Locust bean gum: Processing, properties and food applications—A review. Int. J. Biol. Macromol..

[4] Karabulut A., Canbolat O., Kamalak A. (2006). Evaluation of carob, Ceratonia siliqua pods as a feed for sheep. Livest. Res. Rural Dev..

[5] Nasar-Abbas S.M., e-Huma Z., Vu T.-H., Khan M.K., Esbenshade H., Jayasena V. (2016). Carob kibble: A bioactive-rich food ingredient. Compr. Rev. Food Sci. Food Saf..

[6] Goulas V., Stylos E., Chatziathanasiadou M.V., Mavromoustakos T., Tzakos A.G. (2016). Functional components of carob fruit: Linking the chemical and biological space. Int. J. Mol. Sci..

[7] Galanakis C.M. (2018). Phenols recovered from olive mill wastewater as additives in meat production. Trends Food Sci. Technol..

[8] Sakakibara H., Honda Y., Nakagawa S., Ashida H., Kanazawa K. (2003). Simultaneous determination of all polyphenols in vegetables, fruits, and teas. J. Agric. Food Chem..

[9] Youssef M.K.E., El-Manfaloty M.M., Ali H.M. (2013). Assessment of proximate chemical composition, nutritional status, fatty acid composition and phenolic compounds of carob (*Ceratonia siliqua* L.). Food Public Health.

[10] Almanasrah M., Roseiro L.B., Bogel-Lukasik R., Carvalheiro F., Brazinha C., Crespo J., Kallioinen M., Mänttäri M., Duarte L.C. (2015). Selective recovery of phenolic compounds and carbohydrates from carob kibbles using water-based extraction. Ind. Crops Prod..

[11] Rtibi K., Selmi S., Grami D., Amri M., Eto B., El-benna J., Sebai H., Marzouki L. (2017). Chemical constituents and pharmacological actions of carob pods and leaves (*Ceratonia siliqua* L.) on the gastrointestinal tract: A review. Biomed. Pharmacother..

[12] Roseiro L.B., Tavares C.S., Roseiro J.C., Rauter A.P. (2013). Antioxidants from aqueous decoction of carob pods biomass (*Ceretonia siliqua* L.): Optimisation using response surface methodology and phenolic profile by capillary electrophoresis. Ind. Crops Prod..

[13] Stavrou I.J., Christou A., Kapnissi-Christodoulou C.P. (2018). Polyphenols in carobs: A review on their composition, antioxidant capacity and cytotoxic effects, and health impact. Food Chem..

[14] Chan C.-H., Yusoff R., Ngoh G.-C., Kung F.W.-L. (2011). Microwave-assisted extractions of active ingredients from plants. J. Chromatogr. A.

[15] Routray W., Orsat V. (2012). Microwave-assisted extraction of flavonoids: A review. Food Bioproc. Technol..

[16] Baş D., Boyaci İ.H. (2007). Modeling and optimization I: Usability of response surface methodology. J. Food Eng..

[17] Zeković Z., Vladić J., Vidović S.E., Adamović D., Pavlić B. (2016). Optimization of microwave-assisted extraction (MAE) of coriander phenolic antioxidants—response surface methodology approach. J. Sci. Food Agric..

[18] Huma Z.E., Jayasena V., Nasar-Abbas S.M., Imran M., Khan M.K. (2018). Process optimization of polyphenol extraction from carob (*Ceratonia siliqua*) kibbles using microwave-assisted technique. J. Food Process. Preserv..

[19] Quiles-Carrillo L., Mellinas C., Garrigos M.C., Balart R., Torres-Giner S. (2019). Optimization of microwave-assisted extraction of phenolic compounds with antioxidant activity from carob pods. Food Anal. Methods.

[20] Teslić N., Bojanić N., Rakić D., Takači A., Zeković Z., Fišteš A., Bodroža-Solarov M., Pavlić B. (2019). Defatted wheat germ as source of polyphenols—Optimization of microwave-assisted extraction by RSM and ANN approach. Chem. Eng. Process..

[21] Al-Olayan E.M., El-Khadragy M.F., Alajmi R.A., Othman M.S., Bauomy A.A., Ibrahim S.R., Moneim A.E. (2016). *Ceratonia siliqua* pod extract ameliorates *Schistosoma mansoni*-induced liver fibrosis and oxidative stress. BMC Complement. Altern. Med..

[22] Rtibi K., Selmi S., Jabri M.A., El-Benna J., Amri M., Marzouki L., Sebai H. (2016). Protective effect of *Ceratonia siliqua* L. against a dextran sulfate sodium-induced alterations in liver and kidney in rat. J. Med. Food.

[23] Espín J.C., Soler-Rivas C., Wichers H.J. (2000). Characterization of the total free radical scavenger capacity of vegetable oils and oil fractions using 2,2-diphenyl-1-picrylhydrazyl radical. J. Agric. Food Chem..

[24] Oyaizu M. (1986). Studies on products of browning reaction. Antioxidative activities of products of browning reaction prepared from glucosamine. Jpn. J. Nutr. Diet..

[25] Re R., Pellegrini N., Proteggenete A., Pannala A., Yang M., Rice-Evans C. (1999). Antioxidant activity applying an improved ABTS radical cation decolorization assay. Free Radic. Biol. Med..

[26] Derringer G. (1980). Simultaneous optimization of several response variables. J. Qual. Technol..

[27] Salaj N., Kladar N., Srđenović Č.B., Jeremić K., Barjaktarović J., Hitl M., Gavarić N., Božin B. (2020). Stabilization of sunflower and olive oils with savory (*Satureja kitaibelii,* Lamiaceae). J. Food Nutr. Res..

[28] Rašković A., Ćućuz V., Torović L., Tomas A., Gojković-Bukarica L., Ćebović T., Milijašević B., Stilinović N., Hogervorst J.C. (2019). Resveratrol supplementation improves metabolic control in rats with induced hyperlipidemia and type 2 diabetes. Saudi Pharm. J..

[29] Buege J.A., Aust S.D. (1978). Microsomal lipid peroxidation. Meth. Enzymol..

[30] McCord J.M., Fridovich I. (1969). Superoxide dismutase: An enzymic function for erythrocuprein (hemocuprein). J. Biol. Chem..

[31] Liyana-Pathirana C., Shahidi F. (2005). Optimization of extraction of phenolic compounds from wheat using response surface methodology. Food Chem..

[32] Beers R.F., Sizer I.W. (1952). A spectrophotometric method for measuring the breakdown of hydrogen peroxide by catalase. J. Biol. Chem..

[33] Beutler E. (1984). Red Cell Metabolism: A Manual of Biochemical Methods.

[34] Goldberg D.M., Spooner R.J., Bergmeyen H.V. (1983). Assay of Glutathione Reductase. Methods of Enzymatic Analysis.

[35] Sharma R., Watson R.R., Preedy V.R., Zibadi S. (2014). Polyphenols in health and disease: Practice and mechanisms of benefits. Polyphenols in Human Health and Disease.

[36] Roseiro L.B., Duarte L.C., Oliviera D.L., Roque R., Bernardo-Gil M.G., Martins A.I., Sepúlveda C., Almeida J., Meireles M., Gírio F.M. (2013). Supercritical, ultrasound and conventional extracts from carob (*Ceratonia siliqua* L.) biomass: Effect on the phenolic profile and antiproliferative activity. Ind. Crops Prod..

[37] Bernardo-Gil M.G., Roque R., Roseiro L.B., Duarte L.C., Gírio F., Esteves P. (2011). Supercritical extraction of carob kibbles (*Ceratonia siliqua* L.). J. Supercrit. Fluids.

[38] Goulas V., Georgiou E. (2020). Utilization of carob fruit as sources of phenolic compounds with antioxidant potential: Extraction optimization and application in food models. Foods.

[39] Rašković A., Bukumirović N., Paut Kusturica M., Milić N., Čabarkapa V., Borišev I., Čapo I., Miljković D., Stilinović N., Mikov M. (2019). Hepatoprotective and antioxidant potential of Pycnogenol^®^ in acetaminophen-induced hepatotoxicity in rats. Phytother. Res..

[40] Ahmed M.M. (2010). Biochemical studies on nephroprotective effect of carob (*Ceratonia siliqua* L.) growing in Egypt. Nat. Sci..

[41] Suzek H., Celik I., Dogan A. (2017). Nephroprotective hepatoprotective potential and antioxidant role of carob pods (*Cerotonia siliqua* L.) against carbon tetrachloride-induced toxicity in rats. Indian J. Pharm. Educ. Res..

[42] Stilinović N., Čapo I., Vukmirović S., Rašković A., Tomas A., Popović M., Sabo A. (2020). Chemical composition, nutritional profile and *in vivo* antioxidant properties of the cultivated mushroom *Coprinus comatus*. R. Soc. Open Sci..

[43] Hsouna A.B., Saoudi M., Trigui M., Jamoussi K., Boudawara T., Jaoua S., El Feki A. (2011). Characterization of bioactive compounds and ameliorative effects of *Ceratonia siliqua* leaf extract against CCl_4_ induced hepatic oxidative damage and renal failure in rats. Food Chem. Toxicol..

[44] Souli A., Sebai H., Chehimi L., Rtibi K., Tounsi H., Boubaker S., Sakly M., El-Benna J., Amri M. (2015). Hepatoprotective effect of carob against acute ethanol-induced oxidative stress in rat. Toxicol. Ind. Health.

[45] Rtibi K., Jabri M.A., Selmi S., Souli A., Sebai H., El-Benna J., Amri M., Marzouki L. (2015). Gastroprotective effect of carob (*Ceratonia siliqua* L.) against ethanol-induced oxidative stress in rat. BMC Complement. Altern. Med..

[46] Pandit A., Sachdeva T., Bafna P. (2012). Drug-induced hepatotoxicity: A review. J. Appl. Pharm. Sci..

[47] Akakpo J.Y., Ramachandran A., Curry C.S., Rumack B.H., Jaeschke H. (2022). Comparing N-acetylcysteine and 4-methylpyrazole as antidotes for acetaminophen overdose. Arch. Toxicol..

[48] Woolbright B.L., Jaeschke H. (2017). Role of the inflammasome in acetaminophen-induced liver injury and acute liver failure. J. Hepatol..

[49] Mossanen J.C., Krenkel O., Ergen C., Govaere O., Liepelt A., Puengel T., Heymann F., Kalthoff S., Lefebvre E., Eulberg D. (2016). Chemokine (C-C motif) receptor 2-positive monocytes aggravate the early phase of acetaminophen-induced acute liver injury. Hepatology.

[50] Rahman N., Pervin M., Kuramochi M., Karim M.R., Izawa T., Kuwamura M., Yamate J. (2018). M1/M2-macrophage polarization-based hepatotoxicity in d-galactosamine-induced acute liver injury in rats. Toxicol. Pathol..

[51] Frodermann V., Nahrendorf M. (2018). Macrophages and cardiovascular health. Physiol. Rev..

[52] Yamate J., Izawa T., Kuwamura M. (2016). Histopathological analysis of rat hepatotoxicity based on macrophage functions: In particular, an analysis for thioacetamide-induced hepatic lesions. Food Saf..

[53] Tosello-Trampont A.C., Landes S.G., Nguyen V., Novobrantseva T.I., Hahn Y.S. (2012). Kuppfer cells trigger nonalcoholic steatohepatitis development in diet-induced mouse model through tumor necrosis factor-α production. J. Biol. Chem..

