# Supplementary material for: Hepatoprotective Effect of Carob Pulp Flour (Ceratonia siliqua L.) Extract Obtained by Optimized Microwave-Assisted Extraction
Source: Pharmaceutics. 2022 Mar 17;14(3):657. doi: 10.3390/pharmaceutics14030657 (PMC8950939; doi:10.3390/pharmaceutics14030657)
Supplement: Supplementary file 1 [file pharmaceutics-14-00657-s001.zip › pharmaceutics-1611703-supplementary.pdf]

pharmaceutics

Supplementary Materials: Hepatoprotective Effect of Carob Pulp Flour (*Ceratonia siliqua* L.) Extract Obtained by Optimized Microwave-Assisted Extraction

Nikola Martić, Jana Zahorec, Nebojša Stilinović, Bojana Andrejić-Višnjić, Branimir Pavlić, Nebojša Kladar, Dragana Šoronja-Simović, Zita Šereš, Miodrag Vujčić, Olga Horvat and Aleksandar Rašković

Table S1. Animal body mass (mean ± SD; n=7) in animals treated with saline and carob extract.

	ConS	ConP	CE100	CE100+P	CE200	CE200+P
BM (g)	37.00 ± 1.41	37.86 ± 3.08	32.43 ± 2.88	39.86 ± 3.67	34.14 ± 2.97	37.71 ± 2.06
